# Running economy and lower extremity stiffness in endurance runners: A systematic review and meta-analysis

**DOI:** 10.3389/fphys.2022.1059221

**Published:** 2022-11-28

**Authors:** Bowen Liu, Jinlong Wu, Qiuqiong Shi, Fengwei Hao, Wen Xiao, Jingxuan Yu, Fengyu Yu, Zhanbing Ren

**Affiliations:** ^1^ College of Physical Education, Shenzhen University, Shenzhen, China; ^2^ College of Physical Education, Southwest University, Chongqing, China; ^3^ Institute of Textiles and Clothing, The Hong Kong Polytechnic University, Hong Kong, China; ^4^ School of Physical Education and Sports Exercise, South China Normal University, Guangzhou, China

**Keywords:** spring, vertical stiffness, leg stiffness, joint stiffness, running economy

## Abstract

**Background:** Lower extremity stiffness simulates the response of the lower extremity to landing in running. However, its relationship with running economy (RE) remains unclear. This study aims to explore the relationship between lower extremity stiffness and RE.

**Methods:** This study utilized articles from the Web of Science, PubMed, and Scopus discussing the relationships between RE and indicators of lower extremity stiffness, namely vertical stiffness, leg stiffness, and joint stiffness. Methodological quality was assessed using the Joanna Australian Centre for Evidence-Based Care (JBI). Pearson correlation coefficients were utilized to summarize effect sizes, and meta-regression analysis was used to assess the extent of this association between speed and participant level.

**Result:** In total, thirteen studies involving 272 runners met the inclusion criteria and were included in this review. The quality of the thirteen studies ranged from moderate to high. The meta-analysis results showed a negative correlation between vertical stiffness (r = −0.520, 95% CI, −0.635 to −0.384, *p* < 0.001) and leg stiffness (r = −0.568, 95% CI, −0.723 to −0.357, *p* < 0.001) and RE. Additional, there was a small negative correlation between knee stiffness and RE (r = −0.290, 95% CI, −0.508 to −0.037, *p* = 0.025). Meta-regression results showed that the extent to which leg stiffness was negatively correlated with RE was influenced by speed (coefficient = −0.409, *p* = 0.020, *r* ^2^ = 0.79) and participant maximal oxygen uptake (coefficient = −0.068, *p* = 0.010, *r* ^2^ = 0.92).

**Conclusion:** The results of this study suggest that vertical, leg and knee stiffness were negatively correlated with RE. In addition, maximum oxygen uptake and speed will determine whether the runner can take full advantage of leg stiffness to minimize energy expenditure.

## Introduction

Running is a popular sport around the world. Statistics from the US government show that more than 55.9 million people in 2017 were involved in running, jogging, and trail running (Statista, 2018). Khammassi et al. (2020) state that these regular, high-intensity physical activities offer many health benefits. In another study, Ezzatvar et al. (2022) stated that running can reduce the risk of infection and improve immune function, especially during pandemics such as the COVID-19 pandemic. Although running is considered an effective approach to maintain the health of the body, runners use it to enhance their athletic performance. There are many complex factors affecting the performance of runners. These factors include maximum oxygen uptake, lactate threshold and running economy (Joyner, 1991). Running economy (RE) seems to be the most appropriate parameter to distinguish between the endurance running performance of untrained and trained runners (Conley and Krahenbuhl, 1980; Williams and Cavanagh, 1987; Santos-Concejero et al., 2014). Running economy is the oxygen uptake at a given submaximal running speed (Conley and Krahenbuhl, 1980; Morgan and Craib, 1992; Anderson, 1996). Some studies define RE as the energy cost per unit distance (Rodrigo-Carranza et al., 2021). Running economy is one of the most important factors in determining endurance running performance (di Prampero et al., 1986).

When a person is running, bones, muscles, tendons, ligaments and other elements are usually modeled as Spring Mass Model (SMM) to support the body running forward (Blickhan, 1989; Butler et al., 2003). When a person is running, sufficient lower extremity stiffness is required to maintain motor performance. The mechanical stiffness of the lower extremity may be reflected by the vertical, leg, and joint stiffness (McMahon and Cheng, 1990; Butler et al., 2003).

Vertical stiffness describes the vertical displacement of the center of mass (COM) in response to vertical ground reaction force during a task performed in the sagittal plane (Latash and Zatsiorsky, 1993). The vertical stiffness is considered the first stiffness parameter to be measured, and the models for leg and joint stiffness were expanded (Morin et al., 2005; Brughelli and Cronin, 2008). The measurement of vertical stiffness requires the least amount of equipment in the experiment and can be obtained fast (Maloney and Fletcher, 2021). However, the calculation of vertical stiffness only attempts to model the cumulative stiffness of the lower extremity holistically and does not consider the effects of specific details. Leg stiffness characterizes the structural components of the leg, including muscles, tendons, and ligaments (Kerdok et al., 2002), which are an important component of the stretch-shortening cycle (SSC). These structural components of the leg reflect the compression of the leg spring in any plane or direction corresponding to the force (McMahon and Cheng, 1990; Hunter and Smith, 2007). During running, the leg touches the ground at a certain angle (*θ*). Therefore, the leg spring compression is greater than the COM displacement. Some studies have found that the leg stiffness is always less than the vertical stiffness (Farley and González, 1996; Matt; Brughelli and Cronin, 2008). Also, when the theta angle is 0 during vertical jumping, it leads to a leg stiffness that produces the same value as the vertical stiffness (Beerse and Wu, 2017). Many researchers refer to the results calculated using the equation for vertical stiffness as leg stiffness, which may cause confusion among readers about this concept (Maloney and Fletcher, 2021; Struzik et al., 2021).

The vertical stiffness and leg stiffness were calculated using the whole lower extremity as a whole SMM (Blickhan, 1989; Butler et al., 2003), and such measurements do not take into account multiple degrees of freedom of the lower extremity. Therefore, Farley and his colleagues proposed a joint stiffness calculated with the torsion spring model. First, the researchers deconstructed the hip, knee and ankle joints into three torsion springs (Farley et al., 1998). Then, the relative contribution of the stiffness of the three joints to the overall leg spring stiffness was evaluated (Farley et al., 1998). Joint stiffness describes the resistance to changes in angular displacement in flexion and rotation after applying a joint moment (Latash and Zatsiorsky, 1993; Butler et al., 2003; Brughelli and Cronin, 2008). The inverse dynamics principle obtains this net joint moment. By using a torsional spring model, joint stiffness values can be estimated for the hip, knee and ankle joints during vertical and horizontal motion. Previously, neither the vertical nor the leg stiffness could detect how each joint relative contributes to the stiffness of the whole leg. Joint stiffness in a running task can be used to consider the relative contribution of each joint to total stiffness (Butler et al., 2003; Maloney and Fletcher, 2021).

Researchers started exploring the relationship between stiffness and energy expenditure in the 1990s. However, there is still no higher quality evidence for the relationship between lower extremity stiffness and energy expenditure, and some studies have even found opposite results for each other (Tam et al., 2018; Zhang et al., 2022). Furthermore, no scholar has yet reviewed specifically the relationship between lower extremity stiffness and RE. If lower extremity stiffness is related to RE, these results may be a key indicator to evaluate the exercise performance of endurance runners. The purpose of this study was to explore whether lower extremity stiffness was associated with RE in endurance runners.

## Methods

This review followed the Preferred Reporting Items for Systematic Review and Meta-Analyses (PRISMA) statement for reporting systematic reviews.

### Search strategy

The literature search was conducted on three databases, PubMed, Web of Science, and Scopus. Studies published between 1980 and 2022 were screened. Journal article titles, abstracts, and keywords in each database were searched using the following terms and Boolean operators: (running economy OR energy cost OR metabolic cost OR energetics OR VO_2_ OR VO_2max_ OR cost of running OR consumed oxygen OR oxygen uptake) AND (leg OR lower limb OR lower extremity OR vertical OR joint) AND (stiffness).

The study was conducted in two stages. First, two independent reviewers evaluated and included potential studies based on titles and abstracts. Second, the selected research papers were categorized as meeting the inclusion criteria (yes), likely to be included (maybe) and not meeting the inclusion criteria (no). Finally, disagreements between the two independent reviewers were discussed and resolved in a consensus session. A third reviewer was consulted where consensus could not be reached.

### Inclusion criteria and data extraction

The articles included in this study met the following criteria: 1) Healthy middle-distance runners aged >18 years with a maximal oxygen uptake (VO_2max_) > 50 ml/kg/min. 2) The study included the observation of both RE and lower extremity stiffness metrics. 3) Correlations between lower-extremity stiffness and RE, such as Pearson’s correlation coefficient or r-square, are reported.

On the other hand, papers were excluded if they 1) were conference presentations, posters, or case studies; 2) included non-runners (e.g., athletes in basketball, and soccer); 3) did not provide data or inferred data on RE or biomechanical characteristics; and 4) did not report relevance.

After the full-text screening, a custom table was created to record basic information about the study (e.g., age, country, height, VO_2max_, and test speed). In addition, Pearson’s correlation coefficient (*r*) was extracted for the relevant stiffness parameter and energy expenditure. For studies reporting r-square, the square root of the coefficient of determination was taken by its directionality and was transformed into a correlation coefficient.

### Quality evaluation

The methodological quality of each study was assessed using the Joanna Briggs Institute’s (JBI) Analytical Cross-Sectional Study Critical Assessment Tool (Moola et al., 2017). The checklist consists of ten items to evaluate the suitability of studies for inclusion in systematic reviews and meta-analyses. Two assessors assigned each study a score of 2 for “yes,” 0 for “no,” and 1 for “unclear.” Studies with a total score greater than 70% were considered to be of high quality.

### Data analysis

A comprehensive meta-analysis version 3.0 (Englewood, NJ, United States) was used to analyze pooled Pearson’s correlation coefficients. A *p*-value of less than 0.05 was considered significantly different. The correlation coefficient was used to summarize results and report 95% confidence intervals. The heterogeneity of studies was assessed using the *I* ^2^ statistic. Studies with *I* ^2^ of less than 50% had poor heterogeneity, and a fixed-effects model was considered; otherwise, a random-effects model was used (Buccheri et al., 2018). According to Hopkins evaluation scheme, correlation size was judged as follows: <0.1 = trivial, 0.1–0.29 = small, 0.30–0.49 = moderate, 0.50–0.69 = large, 0.70–0.89 = very large, and ≥0.90 = extremely large (Hopkins et al., 2009). A simple meta-regression analysis was performed on the two independent variables, VO_2max_ and test speed, to determine the variation of the moderating variables on the magnitude of the correlation. The correlation coefficients for each study were converted to Fisher’s Z-score as the dependent variable for the regression analysis. To assess the effect of individual studies on the pooled correlation coefficients and to test the robustness of the correlation between running economy and lower extremity stiffness, sensitivity analyses were performed by sequentially reducing one study per round. Publication bias was examined using funnel plots, and Duval and Tweedie’s trim and fill correction were used to address the impact of publication bias on the main meta-analysis.

## Result

### Study identification and characteristics

**FIGURE 1:**
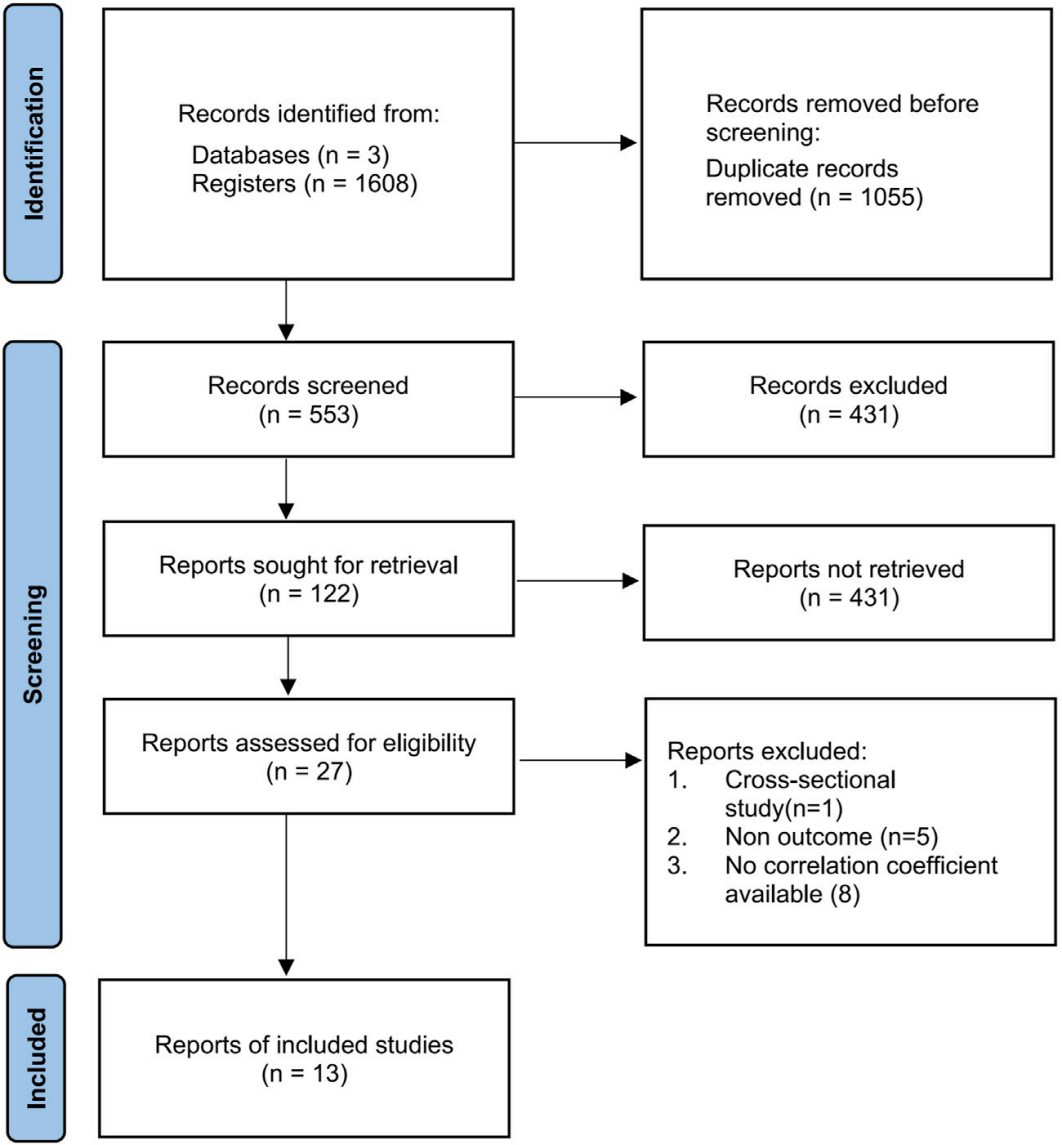
PRISMA flow diagram showing the selection process of eligible articles.

In total, 1,608 articles were obtained by searching three databases including PubMed, Web of Science, and Scopus. The Endnote software was used to remove duplicates, and 553 articles remained. In addition, papers with only titles and abstracts were filtered, and 27 articles with full texts remained. These articles were further screened. Out of the 27 articles, one longitudinal article included an intervention category, five did not include energy consumption or stiffness metrics, and eight did not include an available correlation coefficient. These articles were excluded. As a result, thirteen articles were included in our systematic review (Dalleau et al., 1998; Heise and Martin, 1998; Slawinski et al., 2008; Rabita et al., 2011; Barnes et al., 2014; Lazzer et al., 2014; Man et al., 2016; Rogers et al., 2017; Tam et al., 2018; Monte et al., 2020; Hansen et al., 2021; Li et al., 2021; Zhang et al., 2022), as shown in Figure 1.

In total, 272 runners were included in this study, 90.44% male and 9.56% female, with a mean age of 24.93 ± 6.48 years. The VO_2max_ of the participants included in the study was 54.02–71.5 ml/kg/min. Ten studies reported correlations between vertical stiffness and RE, ten other studies reported correlations between leg stiffness and RE, and two studies reported correlations between knee stiffness and ankle stiffness and RE.

To acquire vertical, leg, and joint stiffness, the majority (*n* = 11) of the studies were conducted during running. Barnes and his colleagues used the peak force measured during five squat jumps divided by the vertical displacement as the “leg stiffness” (Barnes et al., 2014), which we summarized as the vertical stiffness. The approach used for measuring vertical stiffness was adopted by Rogers et al. (2017). In all studies, leg stiffness and joint stiffness were measured during running. Details of the included studies are shown in Table 1.

**TABLE 1:** Characteristics of the included studies.

Author	Country	Participant (male)	Age (years)	VO_2max_ (ml/kg/min	Intensity (m/s)	Unit of measure (RE)
Zhang et al. (2022)	China	30 (30)	20.00–22.00	54.02 ± 4.67	2.78	ml/kg/min
Li et al. (2021)	China	28 (28)	20.70 ± 1.20	65.78 ± 4.99	3.33	ml/kg/min
					3.89	
					4.44	
Hansen et al. (2021)	Denmark	12 (12)	22.40 ± 3.10	67.04 ± 4.2	3.89	ml/kg/min
					5.00	
Monte et al. (2020)	Italy	32 (32)	37.90 ± 13.00	Endurance runners	3.56 ± 0.34	J/kg/m
Tam et al. (2019)	South Africa	30 (30)	258.00 ± 5.00	Trained runners	3.30	ml/kg/min
Rogers et al. (2017)	Australia	11 (11)	20.00 ± 2.90	67.60 ± 3.80	3.89	Kcal/kg/km
Man et al. (2016)	China	9 (9)	20.00 ± 3.90	Experienced runners	2.87	ml/min kg^−0.75^
Lazzer et al. (2014)	Italy	15 (15)	40.50 ± 8.40	55.20 ± 6.70	2.78	ml/kg/m
Barnes et al. (2014)	New	39 (39)	20.80 ± 2.80	68.70 ± 4.80	3.89	ml/kg/min
	Zealand	24 (0)	20.50 ± 2.10	59.90 ± 3.50		
Rabita et al. (2011)	France	9 (6)	23.20 ± 3.20	71.50 ± 6.50	5.10 ± 0.30	J/kg/m
Slawinski et al. (2008)	France	9 (7)	NR	67.80 ± 5.20 (male) < 59.30 ± 4.70 (female)	5.25 ± 0.40	ml/kg/m
Heise et al. (1998)	United States	16 (16)	27.30 ± 4.80	62.20 ± 3.00	3.35	ml/kg/min
Dalleau et al. (1998)	France	8 (8)	24.00 ± 5.00	65.60 ± 4.60	5.10 ± 0.33	ml/kg/m

### Quality evaluation

Table 2 shows the quality evaluation scores for each study (Table 2). The mean JBI quality rating score for the thirteen studies included in the analysis was 15.85 ± 0.86. The majority of these studies (*n* = 12) were of high quality. One study had a low rating because it lacked ethical consideration. A common low-scoring item in the entries was “How was the study population selected?” Given that the purpose of this review was to evaluate a simple correlation between lower extremity stiffness and RE, the sampling and selection of the study population did not affect the results of this study. In particular, most studies had clear objectives, valid and reliable data collection, and appropriate statistical methods. Therefore, no study was excluded because of methodological quality issues.

**TABLE 2:** Methodological quality evaluation for the included studies.

Author	Item 1	Item 2	Item 3	Item 4	Item 5	Item 6	Item 7	Item 8	Item 9	Item 10	Total
Zhang et al. (2022)	2	0	0	2	2	2	2	2	2	1	15
Li et al. (2021)	2	0	0	2	2	2	2	2	2	2	16
Hansen et al. (2021)	2	0	0	2	2	2	2	2	2	1	15
Monte et al. (2020)	2	0	0	2	2	2	2	2	2	2	16
Tam et al. (2017)	2	0	0	2	2	2	2	2	2	2	16
Rogers et al. (2017)	2	0	0	2	2	2	2	2	1	2	15
Man et al. (2016)	2	1	1	1	2	2	2	2	2	2	17
Lazzer et al. (2014)	2	0	2	2	2	2	2	2	2	1	17
Barnes et al. (2014)	1	1	0	2	2	2	2	2	2	2	16
Rabita et al. (2011)	2	1	0	2	2	2	2	2	2	1	16
Slawinski et al. (2008)	2	0	0	2	2	2	2	2	2	2	16
Heise et al. (1998)	2	1	0	2	2	2	0	2	2	1	14
Dalleau et al. (1998)	2	1	0	2	2	2	2	2	2	2	17

Item 1: Is the research purpose of the study clear? Are the arguments sufficient?

Item 2: How was the study population selected? (whether the research subjects were randomly selected and whether stratified sampling was adopted to improve the representativeness of the sample).

Item 3: Are the inclusion and exclusion criteria for the sample clearly described?

Item 4: Are the sample characteristics clearly described?

Item 5: Are the data collection tools reliable and valid? (If an investigator survey is taken, how reproducible is the survey result?)

Item 6: What are the measures to verify the authenticity of the data?

Item 7: Are ethical issues considered?

Item 8: Is the statistical method correct?

Item 9: Is the presentation of the findings appropriate? (Are the results and inferences distinguishable, and are the results faithful to the data rather than inferences).

Item 10: Is the research value clearly articulated?

### Pooled analyses

**FIGURE 2:**
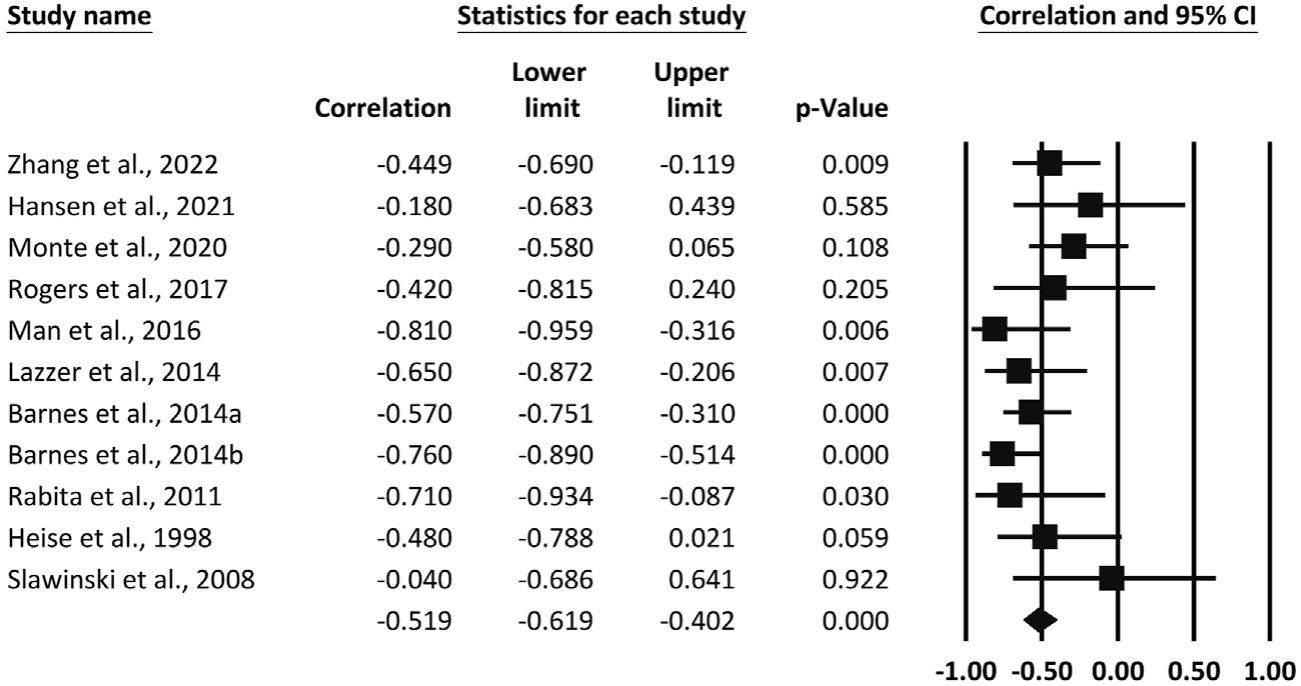
Forest plot of the correlation between vertical stiffness and running economy. The superscripted letter in column 1 refer to speed or gender items assessed in the same study.

**FIGURE 3:**
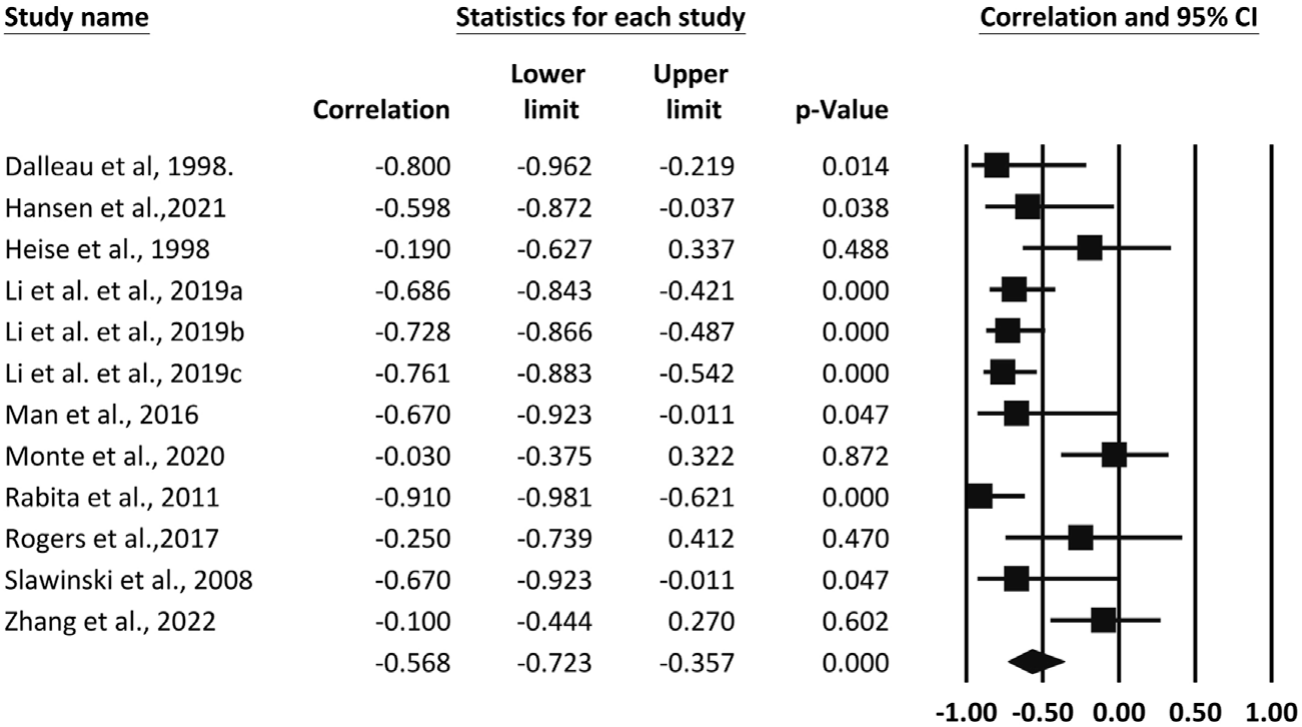
Forest plot of the correlation between leg stiffness and running economy. The superscripted letter in column 1 refer to speed or gender items assessed in the same study.

Ten studies reported Pearson correlation coefficients between vertical stiffness and RE. Heterogeneity between vertical stiffness and RE across studies was small (*I* ^2^ = 20.62%). As a result, a meta-analysis was performed using a fixed effects model. The results revealed a large pooled correlation between vertical stiffness and RE (*r* = -0.519, 95% CI, −0.619 to −0.402, *p* < 0.001), as shown in Figure 2. Ten studies reported Pearson correlation coefficients between leg stiffness and RE. The test for heterogeneity between leg stiffness and RE was moderate (*I* ^2^ = 67.93%). As a result, a random-effects model was adopted for meta-analysis. Pooled results showed a large pooled correlation between leg stiffness and RE (*r* = −0.568, *p* < 0.001, 95% CI, −0.723 to −0.357), as shown in Figure 3.

**FIGURE 4:**
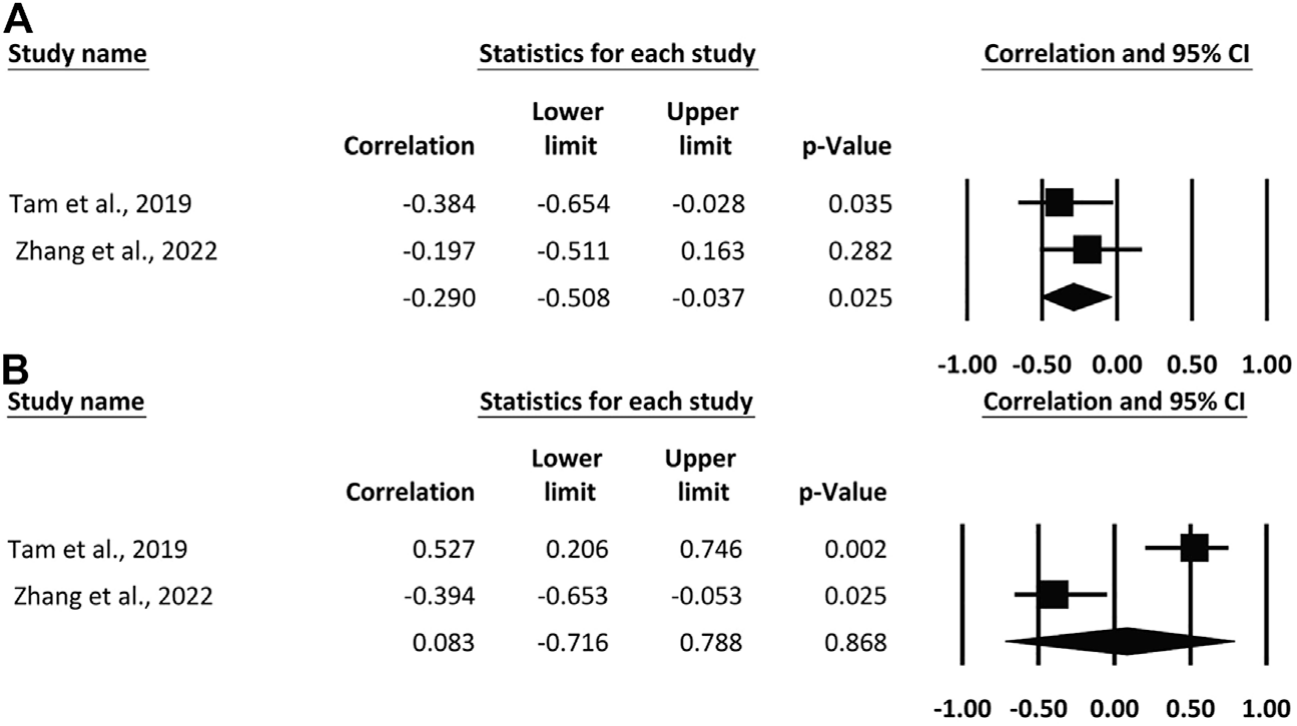
Forest plot of the correlation between knee **(A)** and ankle stiffness **(B)** and running economy.

Two studies reported pooled results for knee stiffness and ankle stiffness. Pooled results with small heterogeneity showed a small negative correlation between knee stiffness and RE (*r* = −0.290, 95% CI, −0.508 to −0.037, *p* = 0.025). In contrast, ankle stiffness was not associated with RE (*r* = 0.0838, 95% CI, −0.716 to 0.788, *p* = 0.86), and interestingly, the two studies presented opposite results. As shown in Figure 4.

**FIGURE 5:**
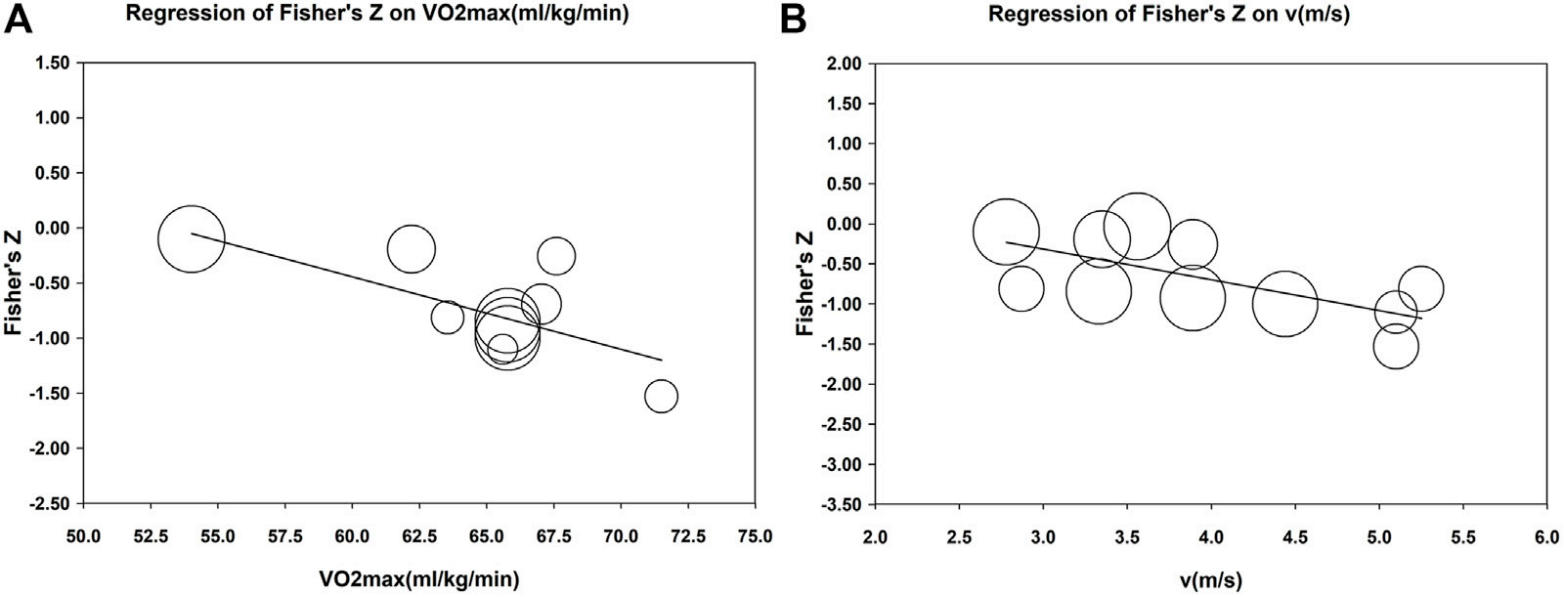
Meta-regression of VO_2max_ **(A)** and test velocity **(B)** with correlation coefficients of leg stiffness and RE. The size of each circle is proportional to the study’s weight.

Further one-way meta-regression analyses were performed for studies to provide clear reporting of test velocity and participant VO_2max_. The results showed that the correlation between vertical stiffness and RE was not affected by participant motion VO_2max_ (coefficient = 0.003, *p* = 0.854) and test velocity (coefficient = 0.046, *p* = 0.751). However, the negative correlation between leg stiffness and RE increased with the VO_2max_ (coefficient = −0.068, 95% CI, −0.114 to −0.022, *p* = 0.010, *r* ^2^ = 0.92) test velocity (coefficient = −0.409, 95% CI, −0.730 to −0.089, *p* = 0.020, *r* ^2^ = 0.79), as shown in Figure 5.

### Sensitivity analyses and publication bias

**FIGURE 6:**
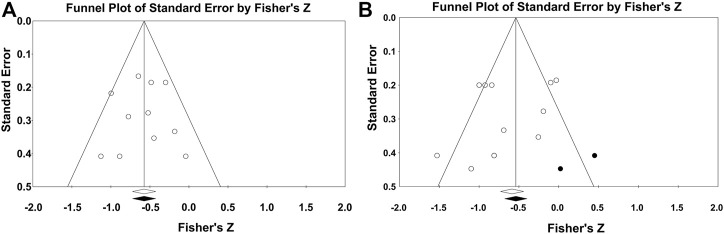
Vertical stiffness **(A)** and leg stiffness **(B)**, funnel plot with running economy correlation.

We performed a sensitivity analysis with one-by-one exclusion and the estimated effects would still be within the 95% CI of the pooled results. Funnel plots of effect sizes versus standard errors were generated to determine possible publication bias. Analysis of the funnel plot revealed that the included studies were generally located on the left side of the pooled results. The funnel plot on the leg stiffness and RE correlations was adjusted using Duval and Tweedie’s trim and fill correction to produce a symmetric funnel plot around the Pearson correlation coefficient. This correction shifted the overall effect size to the right but did not change the main results, which still showed a significant trend, as shown in Figure 6.

## Discussion

This study is the first to examine the relationship between lower extremity stiffness and RE systematically. The aim was to investigate whether lower extremity stiffness affects RE. In total, 13 articles were included in this review. The results showed a significant negative correlation between vertical, and knee stiffness and RE in endurance runners. This result suggests that vertical, leg, and knee stiffness influence RE in endurance runners. In addition, the results of the meta-regression showed that the correlation coefficient between leg stiffness and RE was related to the runner’s speed and VO_2max_.

### Vertical stiffness and running economy

Current findings showed that vertical stiffness negatively correlated with RE during sub-maximal running. The following formula was used to calculate the vertical stiffness:

$$k_{vert}=\frac{F_{\max}}{\Delta y}$$

Where *F*_*max*_ is the peak vertical force, and Δ*y* is the vertical displacement of the COM from ground contact until mid-stance.

Many studies have demonstrated that increasing the peak vertical force or decreasing the COM vertical displacement during running will result in better vertical stiffness. Several studies have found that increasing running speed leads to a higher peak vertical force and a decrease in COM vertical displacement (Matt Brughelli and Cronin, 2008; Brughelli et al., 2011; Halvorsen et al., 2012). Dutto and Sam (2002) found that when the runner is exhausted, the change in vertical stiffness is primarily related to the maximum body displacement in the vertical direction during running rather than the change in peak vertical force. Other studies have also found that runners reduce energy expenditure by exhibiting less COM vertical displacement (Cavanagh et al., 1977; Anderson, 1996; Heise and Martin, 2001; Halvorsen et al., 2012). They observed that the peak vertical force decreases while the vertical stiffness increases significantly with fatigue (Morin et al., 2011; Rabita et al., 2011). This indicated that changing the COM vertical displacement affected the vertical stiffness more than changing the peak vertical force (Cavagna et al., 1988; Morin et al., 2007; Matt; Brughelli and Cronin, 2008). Meanwhile, a positive correlation was found between the vertical displacement of COM and RE (Halvorsen et al., 2012; Tartaruga et al., 2012; Folland et al., 2017). Given the absorption and release of elastic energy, greater vertical oscillation necessarily involves more work against gravity (Moore, 2016; Folland et al., 2017). The first study comparing the biomechanical characteristics of RE in runners at different levels showed that elite runners had fewer vertical oscillations, which were more symmetrical (Cavanagh et al., 1977). Farley et al. (1998) suggested that increasing vertical stiffness by reducing the COM displacement allows the spring-mass system to recoil in a shorter period, facilitating faster absorption and generation of kinetic energy during ground contact. In addition, greater vertical stiffness helped to resist flexion of the lower extremity joint during the support phase. It increased the rate of force generation during the centrifugal (centripetal) phase, which enhanced the storage and utilization of elastic energy during SSC (Matt Brughelli and Cronin, 2008).

The study found that maintaining a high whole-body vertical stiffness minimizes oxygen consumption, and smaller heterogeneity enhances the certainty of the results. Meta-regression results show that the correlation between vertical stiffness and RE is less susceptible to the effects of runner level and speed. Several studies have demonstrated that trained runners who run to exhaustion maintained initial vertical stiffness (Hunter and Smith, 2007; Morin et al., 2011) and even increased it significantly in 24-h ultra-long runs (Morin et al., 2011).

### Leg stiffness and running economy

Our results found the same negative correlation between leg stiffness and RE during the submaximal running, suggesting that leg stiffness is an essential factor influencing RE. Leg stiffness is an essential parameter in regulating running mechanics, which can maintain a stable running gait in humans and animals (Seyfarth et al., 2002). The following equation can calculate the leg stiffness:

$$k_{leg}=\frac{F_{\max}}{∆L},∆L=∆y+L\left(1-\cos\theta \right),\theta =\sin\left(v∙\frac{tc}{2L}\right)$$

where *v* is running velocity and *tc* is contact time. Leg length is symbolized as *L*, which comes into contact with the ground at an angle of *θ* (McMahon and Cheng, 1990).

Several studies support the result of a significant negative correlation in the random effects of this study. Scholars began to focus on the possibility that RE may be related to the time course of supporting body weight, for example, ground contact time (*tc*). Kram et al. (1990) reported that the cost of supporting the animal’s weight and the time course of generating this force determines the cost of running. Because the longer *tc* allows the lower-extremities to generate propulsive force over a longer period of time when in contact with the ground, reducing energy costs (Kram and Taylor, 1990). Notably, many studies on human running showed a significant positive correlation between *tc* and RE (Nummela et al., 2007; Barnes et al., 2014; Di Michele and Merni, 2014; Mooses et al., 2021). This was likely due to less time required for braking to decelerate the body’s forward motion (Nummela et al., 2007; Kong and de Heer, 2008; Mooses et al., 2021). Meanwhile, a negative correlation between leg stiffness and *tc* was demonstrated in other studies (Morin et al., 2007; Hayes and Caplan, 2014; Santos-Concejero et al., 2014; Man et al., 2016). Morin et al. (2007) found that the change in *tc* can account for 90% of the change in leg stiffness. This means that reducing *tc* increases leg stiffness (Man et al., 2016). This may be due to the high preactivation of the calf muscles which then increases the sensitivity of muscle spindle potentiating stretch reflexes to enhance musculo-tendon stiffness and to improve the RE (Santos-Concejero et al., 2014; Mooses et al., 2021). Moreover, Moore et al. (2019) showed that the relationship between leg stiffness and RE is not linear and that there is an identifiable optimum for it, as 90% of runners can keep their optimal metabolic cost within 5% at a self-selected leg stiffness (Moore et al., 2019). Unfortunately, the optimal range of leg stiffness values for endurance runners is still unknown.

The results of the meta-regression explained the high heterogeneity of the correlation between leg stiffness and RE. The results showed that the correlation between leg stiffness and RE increased with the speed and runner VO_2max_. Previous studies showed that novice runners have higher oxygen consumption than trained runners, while *tc* is longer (Williams and Cavanagh, 1987; de Ruiter et al., 2014). Bitchell et al. (2019) state that although leg stiffness was unaffected by physiological training status, untrained runners had difficulty maintaining consistent leg stiffness during running. This variability is likely related to increased oxygen costs. Experienced and well-trained runners optimize elastic energy storage and release more rationally to minimize metabolic costs (Lussiana et al., 2019; Moore et al., 2019).

There has been no consensus whether speed affects leg stiffness in previous studies (Matt Brughelli and Cronin, 2008). Our meta-regression results confirmed that speed enhanced the correlation between leg stiffness and RE. The greater the speed, the more pronounced the favorable effect of leg stiffness on RE. Lai et al. (2014) reported that the energy stored in the elastic deformation of the tendon is better utilized at higher speeds and that the positive work done by the elastic strain energy on the tendon-muscle unit is lower at slower running speeds than at faster running speeds. The results of this study validate previous conjectures that there is indeed a “U” shaped relationship between leg stiffness and RE and that training level and running speed will determine whether a runner’s energy cost is near the bottom of the curve (Zhang et al., 2022).

### Joint stiffness and running economy

The torsional spring model can be utilized to estimate the joint stiffness values of the main joints of the lower extremity during vertical and horizontal movements (Latash and Zatsiorsky, 1993). The following equation can calculate joint stiffness:

$$k_{joint}=\frac{M}{∆\alpha }$$

where *M* denotes the deformation torque and Δα is the deformation angle.

Measurement of joint stiffness, particularly of the ankle and knee joints, provides greater insight into the respective contributions of the joints to the overall stiffness of the lower extremity (Maloney and Fletcher, 2021). The combination of knee and ankle stiffness provides the best correlation for leg stiffness when exploring the variation in leg stiffness (Lorimer et al., 2018). Unfortunately, there is only two evidences supporting the relationship between joint stiffness and RE. Tam et al. (2019) found that high knee and less ankle stiffness were associated with better RE. The results of the meta-analysis showed a negative correlation between knee stiffness and RE, though it was not significant.

Joint stiffness mainly depends on the level of activation of the muscles around the joint (Farley et al., 1998). The most economical runners rely on greater muscle activation (Tam et al., 2019). Kyröläinen et al. (2001) found that muscle co-activation around the knee and ankle joints during running increases joint stiffness, which was associated with better RE. Tam et al. (2017) also showed that during the pre-activation and ground contact, rectus femoris (RD):biceps femoris (BF) co activation ratio was positively correlated with the knee, but not the ankle joint. Pre-emptive neuromuscular joint control decreases the need for corrective muscle activation at and after ground contact, where loading forces are applied to joints stiffness (Tam et al., 2019). This suggested that greater knee stiffness allowed more energy to be stored in the leg spring through co-activation of the agonist and antagonist muscles and provided coordination of the ankle and hip joints (Jin and Hahn, 2018).

Unlike walking, running requires more bracing phase energy generation (Jin and Hahn, 2018). Meanwhile, the ankle joint does more positive work in the support phase than the knee (Jin and Hahn, 2018). During landing, the knee and hip joints absorb more of the impact, which requires higher ankle stiffness and the joint muscle-tendon pull reflex, resulting in more energy demand (Jin and Hahn, 2018). Previous studies have shown that increased gastrocnemius-anterior tibialis activation is associated with high ankle stabilization stability and energy sparing (Kyröläinen et al., 2001; Fletcher and MacIntosh, 2017; Tam et al., 2017; Tam et al., 2019).

The reason for the opposing results in the two studies may stem from differences in landing patterns and running speeds (Tam et al., 2018; Zhang et al., 2022). The forefoot landing ankle stiffness used by the elite runners was lower and performed more negative work (Hamill and Gruber, 2017). This suggests that a high supple joint stiffness absorbs the energy of landing impact to a greater extent. The hindfoot landing has high ankle stiffness and performs less negative function (Hamill and Gruber, 2017). However, as speed decreases and running distance increases, some runners tend to land on their hind feet (Hasegawa et al., 2007). Additionally, Weir et al. (2020) found that habitual hindfoot runners showed a decreased ankle stiffness as the running time increased. In conclusion, the current limited evidence makes the results uncertain and future studies are necessary to address the comparison of ankle stiffness contributions at different running patterns and speeds.

Since the phase shift for the moment-displacement curve of the hip commonly exceeds 10% (Farley and Morgenroth, 1999; Kuitunen et al., 2011; Maloney and Fletcher, 2021), it is often excluded from the comparison. As a result, many studies about joint stiffness have only focused on the knee and ankle joints, and little attention has been paid to hip stiffness. Jin and Hahn (2018) observed that the hip joint plays an important role in energy absorption during the swing phase of running. However, the relationship between hip stiffness and endurance in sports performance is unknown.

### Limitations and prospects

Several limitations were identified in this study. First, the number of studies included in this study was too small, and the Pearson correlation coefficient still has some limitations in expressing the relationship between lower extremity stiffness and RE. Second, the vast majority of subjects in the study were male, with only 11% of females participating. In one of the articles used in this study, women showed a greater correlation than men. Although RE may not differ by gender (Besson et al., 2022), there are differences in the biomechanics of running (Barnes et al., 2014; Besson et al., 2022). For example, women have faster stride length, shorter *tc*, and faster time to peak (Nelson et al., 1977; Williams et al., 1987; Besson et al., 2022). As a result, women have higher leg stiffness than men (Barnes et al., 2014). Besson et al. (2022) stated that male and female runners optimize their running patterns to suit their gender characteristics. In addition, women may have physiological and technical advantages in ultra-endurance events (Tiller et al., 2021). As a result, future researchers need to pay more attention to the relationship between lower extremity biomechanics and energy in runners of different genders.

The above conclusion are based on SMM. This simple model can reproduce the motion and forces of running remarkably well. Schroeder and Kuo (2021) proposed a running model based on SMM that combines elasticity with active actuation and passive dissipation (Actuated Spring-mass model). This model included two types of dissipation, one dissipating for collision and hysteresis losses, and the other including hysteresis to simulate imperfect energy return of tendons and other series elastic tissues (Schroeder and Kuo, 2021). However, the model is not perfect either, for example, it neglects a swing leg, whose active motion may also cost energy (Doke et al., 2005). In the future, more sophisticated models should be adopted to simulate the energy expenditure during running.

Finally, we discussed the relationship between each of the three stiffnesses and RE. A negative correlation was obtained for all except ankle stiffness. However, the consistency results may lead to the mistaken belief that they are independent and interchangeable. They act together on the body in motion and support the body mass. There is a close link between vertical, leg and joint stiffness, for example, the stiffness of the leg spring is influenced by the stiffness of the three joints (hip, knee, and ankle) (Struzik et al., 2021). Therefore, in the future, it is necessary to continue to explore the potential relationships between vertical, leg, and joint.

## Conclusion

This meta-analysis found a significant negative association of vertical, leg, and knee stiffness with RE. Furthermore, the extent of this correlation was related to speed and the runner’s VO_2max_. This suggests that experienced runners make more rational use of elastic energy to minimize energy costs at faster speeds. Some scholars have suggested that there is a weak negative correlation between knee stiffness and oxygen consumption, but it is not clear how much ankle stiffness runners need. Many factors affect stiffness during running, and these factors are influenced by different tasks, populations, and gender. The optimal stiffness required for a runner needs to be further investigated. The results of this study may, to some extent, inform future training and research.

## Data Availability

The original contributions presented in the study are included in the article/Supplementary Material, further inquiries can be directed to the corresponding author.
